# Posterior reversible encephalopathy syndrome in association with exacerbation of chronic obstructive pulmonary disease: a case report

**DOI:** 10.1186/s12883-018-1188-z

**Published:** 2018-10-31

**Authors:** Sushil Khanal, Subhash Prasad Acharya

**Affiliations:** Department of Critical Care, Grande International Hospital (GIH), Kathmandu, Nepal

**Keywords:** Chronic obstructive pulmonary disease, Posterior reversible encephalopathy syndrome, Encephalopathy, Seizure

## Abstract

**Background:**

Posterior reversible encephalopathy syndrome (PRES) is a reversible clinical and neurological entity. There are varieties of comorbid conditions which are associated with PRES. Chronic obstructive pulmonary disease (COPD) is a rare predisposing factor for the development of PRES.

**Case presentation:**

A 55 year old female who was being treated for acute exacerbation of COPD developed altered sensorium and multiple episodes of seizure. Characteristic imaging findings and associated clinical symptoms led us to a diagnosis of PRES in our patient.

**Conclusion:**

Association of PRES and COPD is a rare entity. The diagnosis of PRES should be brought to mind if there is encephalopathy or seizure in COPD exacerbation.

## Background

Posterior Reversible Encephalopathy Syndrome (PRES) is characterized by headache, seizure, altered mental status and visual disturbances. It is a clinical and radiological entity and typically causes reversible changes in the posterior circulation system of the brain [1]. The association of PRES has been described frequently with number of medical conditions like hypertensive encephalopathy, eclampsia, and the use of cytotoxic and immunosuppressant drugs [2]. To the best of our knowledge, there are few case reports of PRES in the background of Chronic obstructive pulmonary disease (COPD). Here, we present a case of a 55 year old female with COPD exacerbation developing the characteristics features of PRES.

## Case presentation

A 55 year old female patient was being treated at local hospital for 3 days symptoms suggestive of acute exacerbation of COPD. She was referred to our center for further management after she developed multiple episodes of seizure followed by loss of consciousness on the first day of hospital admission. The patient’s relatives revealed that she had history of COPD for last 5 years but was not compliant to inhaler medications. Her family history was unremarkable. She has been a smoker for the last 30 years. No other significant history was available.

On examination, the patient was drowsy, and was not obeying commands. She had a temperature of 37.6 °C, blood pressure of 130/80 mmHg, pulse rate of 96/min and respiratory rate of 26/min. She had widespread expiratory wheeze. While the patient was regaining consciousness, she reported of headache and a decreased vision. An ocular examination revealed normally reactive pupil and fundus. Cranial nerves examination was unremarkable. Motor and sensory function examination was normal. There was no any clinical sign of meningeal irritation.

**Fig. 1:**
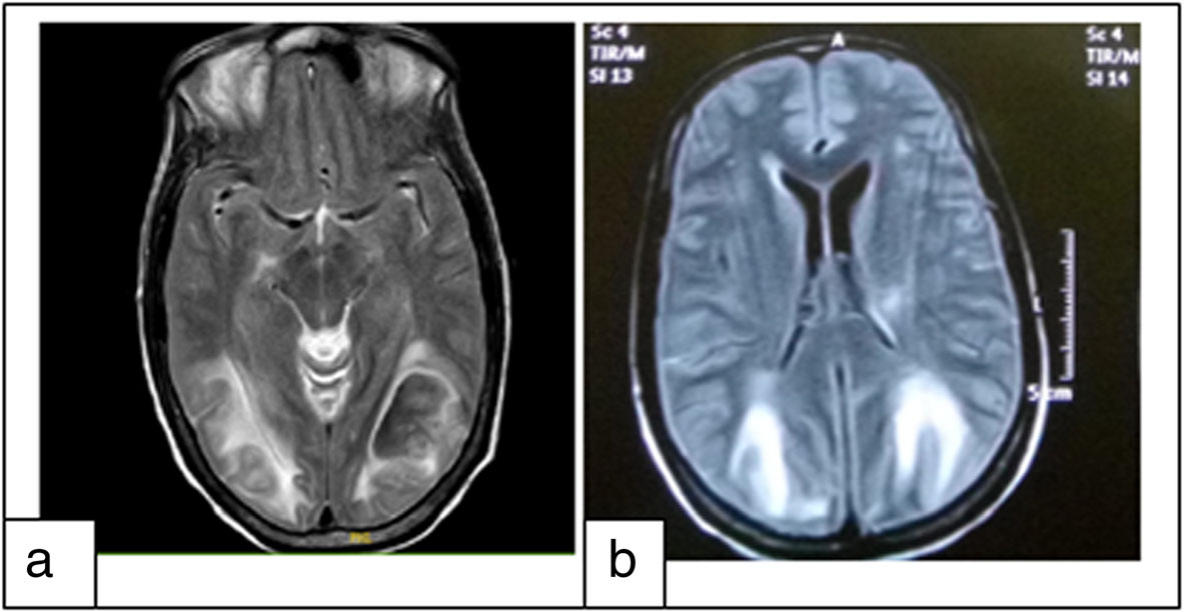
MRI findings (T2 sequence (**a**), FLAIR (**b**)) showing the diffuse confluent white matter hyperintensities in bilateral parieto- occipital region

Her laboratory tests on admission were as follows: hemoglobin, 17 g/dl; white blood cells, 12640 /Cumm; platelets, 155000 /Cumm; urea, 37 mg/ dl; creatinine 0.3 mg/dl; Na, 132 meq/L; K,4.6 meq/L. Chest X-radiography revealed emphysematous changes. Arterial blood gas finding showed the pH, 7.56; pCO_2_, 46.2; pO_2_, 81.0; HCO_3_, 41.5. Magnetic Resonance Imaging (MRI) demonstrated hyperintense lesions in the bilateral parieto-occipital region consistent with PRES (Fig.1).

Our patient was treated with salbutamol and ipratropium nebulisation, hydrocortisone, levetiracetam and other supportive care. The patient was continuously monitored for hemodynamic stability in Intensive Care Unit (ICU) for 2 days and was later transferred to a general ward. The patient continued to improve clinically and was discharged home on the sixth day of hospitalization without any respiratory and neurological symptom.

## Discussion and conclusion

Posterior reversible encephalopathy syndrome (PRES) has been described as a clinical syndrome of headache, altered level of consciousness, visual changes, and seizure. There is a characteristic neuroimaging finding of posterior cerebral white matter edema in PRES. Although pathogenesis of PRES is unclear, it is likely to be the consequences of disordered cerebral autoregulation and endothelial dysfunction [1].

A variety of clinical conditions like hypertensive emergency, renal disease, pre-eclampsia/eclampsia and use of immunosuppressive agents are commonly associated with the development of PRES. There are very few case reports linking the relation of PRES with COPD [3, 4]. Increased level of circulating tumor necrosis factor alpha (TNFα), interleukin-1 (IL-1) and endothelin-1 (ET-1) in COPD causes endothelial dysfunction in cerebral arteries. Infection during COPD exacerbation also raises the levels of IL-1, TNFα and ET-1. This may be the most probable pathophysiology behind the development of PRES during COPD exacerbation [4].

Differential diagnosis of PRES include other neurologic conditions, such as stroke, venous thrombosis, toxic or metabolic encephalopathy, demyelinating disorders, vasculitis, or encephalitis. As there is limited history and broad differential diagnosis, early neuroimaging is crucial for the diagnosis of PRES [5]. Typical MRI findings in PRES are of bilateral white-matter abnormalities in vascular watershed areas in the posterior regions of both cerebral hemispheres, affecting mostly the occipital and parietal lobes [6].

The predilection of posterior brain regions in PRES is not well understood. One possibility involves the regional heterogeneity of the sympathetic innervation of the intracranial arterioles, with better development of sympathetic autoregulation in the anterior circulation than in the posterior circulation [7]. Removal of precipitating factors seem to enhance the full recovery of PRES within a period of days to weeks in most of the cases. However, radiologic improvement lags behind clinical recovery [8, 9].

In Conclusion, there is high rate of admission of COPD in the Intensive Care unit. Although the association of COPD and PRES is rare entity, the differential diagnosis of PRES should be kept in the mind whenever there is encephalopathy or seizure in COPD exacerbation.

## Abbreviations

COPD: Chronic obstructive pulmonary disease
ET-1: Endothelin-1
ICU: Intensive care unit
IL-1: Interleukin-1
MRI: Magnetic resonance imaging
PRES: Posterior reversible encephalopathy syndrome
TNFα: Tumor necrosis factor alpha
